# Aristocracy of Professions

**Published:** 1882-09

**Authors:** J. A. Robinson


### ARISTOCRACY OF PROFESSIONS.

BY J. A. ROBINSON, D.D.S.

The American Dental Association that met at Cincinnati August 1st, had less of the elements of aristocracy than is usually met with at gatherings of this kind. The "divorce case" came up as usual near the close of the meeting, but the general sentiment of the individuality of the profession was so well voiced by Drs. Buckingham and Morgan that the feeling was almost unanimous that dentistry was altogether too large to be made the tail end of the kite of medical practice. If we leave out a little political managing by those who were aspirants for office; and the pedantry and echo of Herbert Spencer, it was a most profitable meeting.

The embryonic season of dentistry has passed, or is fast passing away; and any person visiting the various meetings held all over the continent and seeing the improvements in implements and miniature machinery must be aware of that fact. The old historic dentist of the past, is actually dead, and the real scientific professional gentleman is taking his place. The secrecy observed by leading dentists of the past, necessitated a sort of mechanical plagiarism of all those who were debarred the privilege of entering the profession "by the door;" and so a general iconoclasm became a necessity before the highest ideals of a profession could be reached. The dentist is of necessity an imitator; for the highest types and highest ideals are only imitations of nature. To preserve nature first, and then restore is the function of the dentist. So to imitate, to copy, or form after the highest models is part of dentistry; and the man who is most successful is the best dentist. This reveals the fact to us that thought is simultaneous, and it was manifested by the various contrivances exhibited tending to produce the same results in different ways by persons living many hundreds of miles apart, and who had never seen or thought any other person had seen a similar thing for a similar purpose. As we grow as imitators the need of perfection grows upon us to such an extent that the active mind in striving for the perfect ideal becomes at last a discoverer and then an inventor.

If there is any one place more than another where aristocracy is contemptible it is in associations and conventions.

This villainous compound of self-conceit and vanity wants to be chairman of the committees, or it will not work; it is eternally wasting time in saying the time must not be wasted; button-holing its sycophants to lift it into office; talking loud enough to disturb the meeting while others are speaking, and never listening, however grave the subject or interesting the problem. Without symmetry or strength of understanding it seems like the gaudy sun-flower, yellow and brazen, and calls its *esthetic*, and despises all the violets of thought that bloom modestly in the brain of the unassuming and disdains the words of wisdom that like the scripture rain falls alike on the evil and the good. This is aristocracy.

The formative period of dentistry has also passed—and now the assimilation of the facts discovered are in process of digestion. Empiricism has given place somewhat to philosophical understanding and so we grow. Dr. Atkinson's paper was sharply criticized by Drs. Watt, Rehwinkle and others, who said they listened but could not understand. It may be possible that the vocabulary of Dr. Atkinson may be plain in the future; for Swedenborg who taught more than a hundred years ago was very little understood; but to-day the theories taught by what he called inspiration have entirely remodeled and beautified all the old theologies.

The spirit of love and forbearance largely pervaded the meeting, and that will sustain the creative and imitative energy that is within us till we reproduce the thoughts and manifestations of nature that are alive but latent in the human heart. We must continue to work until philosophy takes the place of empiricism, until assimilation of the past experience of the fathers becomes chyle to nourish and grow us and all future generations.

The divorce case has consolidated the profession, so that prosthetic dentistry has a place where it belongs, viz.: a part of the whole to perfect a system that is necessary for the good of the world. It will not do for the hand to say to the foot I have no need of thee, or the eye to say so of the ear. "All are but parts of one stupendous whole, where body nature is, and God the soul."

The *divorce* (though it may not be acknowledged) grew out of the feeling that many persons engaged in the manufacture of artificial dentures were such poor operators, so illy prepared to perform good work, that even the average dentist felt ashamed to call him by that name, so he was called "cheap John" and "tooth carpenter." But at Cincinnati the prosthetic section was as well received, and called forth as much attention and earnest discussion as any other section.

Dr. Atkinson with his new metallic base; Dr. Buckingham with his experiments in celluloid, a new and simplified celluloid heater; Dr. Robinson with his new metalic filling and lining for rubber and celluloid plates; Dr. Dorrance with his paper on artificial dentures and his new alloy for gold and silver solder were acknowledged as good solid stones in the great divine architecture of dentistry, and were all necessary to hold the superstructure firmly until the dental edifice is finished, and shall be acknowledged an additional monolith whose point shall rise high and shine as a star in the galaxy of professions.

